# Descriptive study of foodborne disease outbreaks using monitoring data from foodborne disease outbreak surveillance system (FDOSS) in Jiaxing City, China, 2013–2023

**DOI:** 10.1186/s12889-025-24287-7

**Published:** 2025-10-06

**Authors:** Yangming Sun, Wei Xu, Yikang Wu, Guoying Zhu, Ping Li, Juanjuan Jia, Hejia Song, Lili Chen, Yun Lin

**Affiliations:** 1 Department of Nutrition and Food Safety, Jiaxing Center for Disease Control and Prevention, 486 Wenqiao Road, Nanhu District, Jiaxing City, Zhejiang Province 314000 China; 2 Department of Environment and Health, Nanhu Center for Disease Control and Prevention, 370 Zhonghuannan Road, Nanhu District, Jiaxing City, Zhejiang Province 314000 China; 3 Department of Nutrition and Food Safety, Zhejiang Provincial Center for Disease Control and Prevention, 3399 Binsheng Road, Binjiang District, Hangzhou City, Zhejiang Province 310051 China

**Keywords:** Foodborne disease outbreak, Poisonous mushroom, *Vibrio parahaemolyticus*

## Abstract

**Background:**

Foodborne diseases are a serious public health problem that cause a heavy burden of illness. We aimed to analyze the characteristics of foodborne disease outbreaks (FBDOs) in Jiaxing and provide scientific support for foodborne disease prevention and control.

**Methods:**

Descriptive epidemiological methods were used to statistically analyze the data reported by seven county-level Centers for Disease Control and Prevention (CDCs) in Jiaxing City through the Foodborne Disease Outbreak Surveillance System (FDOSS) from 2013 to 2023.

**Results:**

During the study period, 128 FBDOs were reported, which resulted in 900 cases, 117 hospitalizations, and one death. Bacteria accounted for the highest proportion of outbreaks (58 outbreaks, 45.31%) and cases (623 cases, 69.22%), followed by poisonous mushrooms (28 outbreaks and 70 cases). Households were the setting for the highest proportion of outbreaks (57.03%). Poisonous mushrooms were the most common single food reported (28 outbreaks, 21.88%), followed by aquatic products (17 outbreaks, 13.28%) and meat and meat products (13 outbreaks, 10.16%). In the 58 bacterial FBDOs, the most important setting was households (20 outbreaks, 34.48%), followed by restaurants (15 outbreaks, 25.86%) and rural banquets (13 outbreaks, 22.41%). Outbreaks caused by poisonous mushrooms occurred mainly in households (27 outbreaks, 96.43%). Different types of bacteria tended to be responsible for outbreaks involving different food categories, e.g., *Vibrio parahaemolyticus* was mainly found in aquatic products (64.71%). From 2018 to 2023, the proportion of outbreaks caused by poisonous mushrooms increased from 22.22 to 76.92%. From 2021, poisonous mushrooms became the predominant factor for FBDOs in Jiaxing City.

**Conclusions:**

Households are the most important settings for FBDOs. Poisonous mushrooms and microbial pathogens are the main factors causing FBDOs. Since 2018, the proportion of FBDOs caused by poisonous mushrooms has been increasing annually. Food safety policies targeting high-risk settings and pathogens identified by surveillance data should be formulated to reduce the risk of FBDOs.

## Introduction

Foodborne diseases are usually infectious or toxic conditions caused by bacteria, viruses, parasites, or chemicals that enter the body through contaminated food or water [1]. Despite the strengthening of food safety regulations and improvements in treatment capabilities, foodborne diseases remain a serious public health problem. The World Health Organization (WHO) estimated that 600 million people worldwide fall ill from eating contaminated food each year, resulting in 420,000 deaths and the loss of 33 million healthy life years [2]. Even in developed countries, foodborne diseases are a serious public health issue. In the United States, 31 known pathogens were responsible for approximately 9 million cases, 56,000 hospitalizations, and 1,300 deaths annually [3]. In 2020, 27 European Union Member States reported 3,086 FBDOs and 20,017 human cases. *Salmonella* remains the most frequently reported causative agent for FBDOs [4]. In the Western Pacific Region, 125 million people get sick and more than 50,000 die every year due to foodborne diseases [5]. From 2003 to 2017, China had reported a total of 19,517 FBDOs, resulting in 235,754 cases, 107,470 hospitalizations, and 1457 deaths [6]. Furthermore, foodborne diseases and their outbreaks have a major influence on social productivity, and they impose a heavy economic burden. In 2013, the United States Department of Agriculture (USDA) Economic Research Service (ERS) estimated that the frequency and severity of foodborne illnesses are responsible for $15.5 billion of the losses annually attributable to medical costs, productivity losses, and economic burden due to death [7]. A more recent report from 2021 suggested that these expenses had risen by 13% to an estimated $17.6 billion per year [7].

Therefore, monitoring FBDOs using a foodborne disease surveillance system is essential for early identification and timely responses to reduce the risk of foodborne diseases [8]. In some developed countries such as the United States, the laboratory-based Foodborne Disease Active Surveillance System (FoodNet) and other surveillance systems have been successfully established and applied for early identification, investigation, and risk reduction of FBDOs [8, 9]. To prevent and control the occurrence of foodborne diseases and protect people’s physical health, the China Food Safety Law was implemented in 2009, and a web-based foodborne disease surveillance platform was established in 2011 [10]. This platform includes the FDOSS, Foodborne Disease Surveillance and Reporting System (FDSRS), National Molecular Traceability Network for Foodborne Diseases (TraNet), and other surveillance systems [10]. The China National Center for Food Safety Risk Assessment (CFSA) maintains and manages the platform for data collection and periodic reporting to the National Health Commission [9]. In the FDSRS, the outbreak warning is triggered by cases of foodborne diseases associated with consumption of causative foods. Centers of Disease Control (CDC) assume statutory responsibilities for epidemiological investigations and hygiene handling in FBDOs. After completing the epidemiological investigation of FBDOs, survey reports and outbreak-related information are submitted to the FDOSS.

Through years of effort, FDOSS has collected a large amount of information on FBDOs. The objectives of this study were to analyze the high-risk foods and risk factors associated with outbreaks in Jiaxing City and to help the government formulate and adjust prevention and control strategies for foodborne diseases.

## Methods

### Definition of outbreaks

A foodborne disease outbreak (FBDO) is defined as two or more cases with similar clinical manifestations wherein epidemiological investigations had confirmed a history of shared exposure to foods or drinks [11]. The identification of FBDOs was mainly based on clinical, epidemiological, and food hygiene investigations and laboratory test data. If specific pathogenic factors were not detected in the collected samples and the results of the epidemiological investigations could not determine the cause, technical experts attempted to determine the unknown causes. In accordance with the National Foodborne Disease Surveillance Manual, China’s FDOSS collects the data for all FBDOs with two or more cases as well as outbreaks involving any deaths.

### Data source

From 2013 to 2023, outbreaks were passively reported to the FDOSS from seven county-level CDCs in Jiaxing City. The working staff in the CDCs in Jiaxing City investigated the FBDOs within their jurisdictions and submitted the epidemiological survey reports to the FDOSS in a uniform format. The reported information from each outbreak included location of the outbreak, date of occurrence, setting, causative pathogen, causative food and its categories, number of cases, hospitalizations, and deaths caused by the outbreak. Other details were collected through the FDOSS. The settings for food preparation or consumption were classified into nine categories: households, restaurants, rural banquets, street stalls, retail food outlets, social meal delivery companies, staff canteens, school canteens, and other locations. The etiology of outbreaks was categorized as bacterial, viral, poisonous mushrooms, biological toxins, and chemical agents. Poisonous mushrooms are a type of fungus that grow in the natural environments and contain multiple toxins. They have a similar appearance to some edible mushrooms and may cause serious poisoning symptoms or even endanger life if ingested by mistake.

### Statistical analysis

The data collected from the FDOSS were analyzed using Excel 2013. Population data for different years were collected from the website of the Jiaxing Bureau of Statistics [12]. Descriptive research methods were used to analyze the seasonal characteristics and the characteristics of the etiology, settings, causative food. R version 4.3.3 was used to conduct Cox-Staurt trend test and Chi-square test, *p* < 0.05 was considered to be statistically significant.

### Ethics statement

This study was conducted in accordance with the Declaration of Helsinki, and approved by the Ethics Committee of Jiaxing Center for Disease Control and Prevention (CDC). The ethics committee approved the procedure for verbal consent because Jiaxing CDC has the authority to collect and utilize information on FBDOs, which is part of disease surveillance scope in Jiaxing CDC. All the participants were notified that they have the right to refuse or terminate the study at any point of the interview. Because we obtained verbal consent, documentation of consent was not required. However, the information provided by each participant was kept confidential in Jiaxing CDC.

## Results

### General epidemiological characteristics

**Fig. 1:**
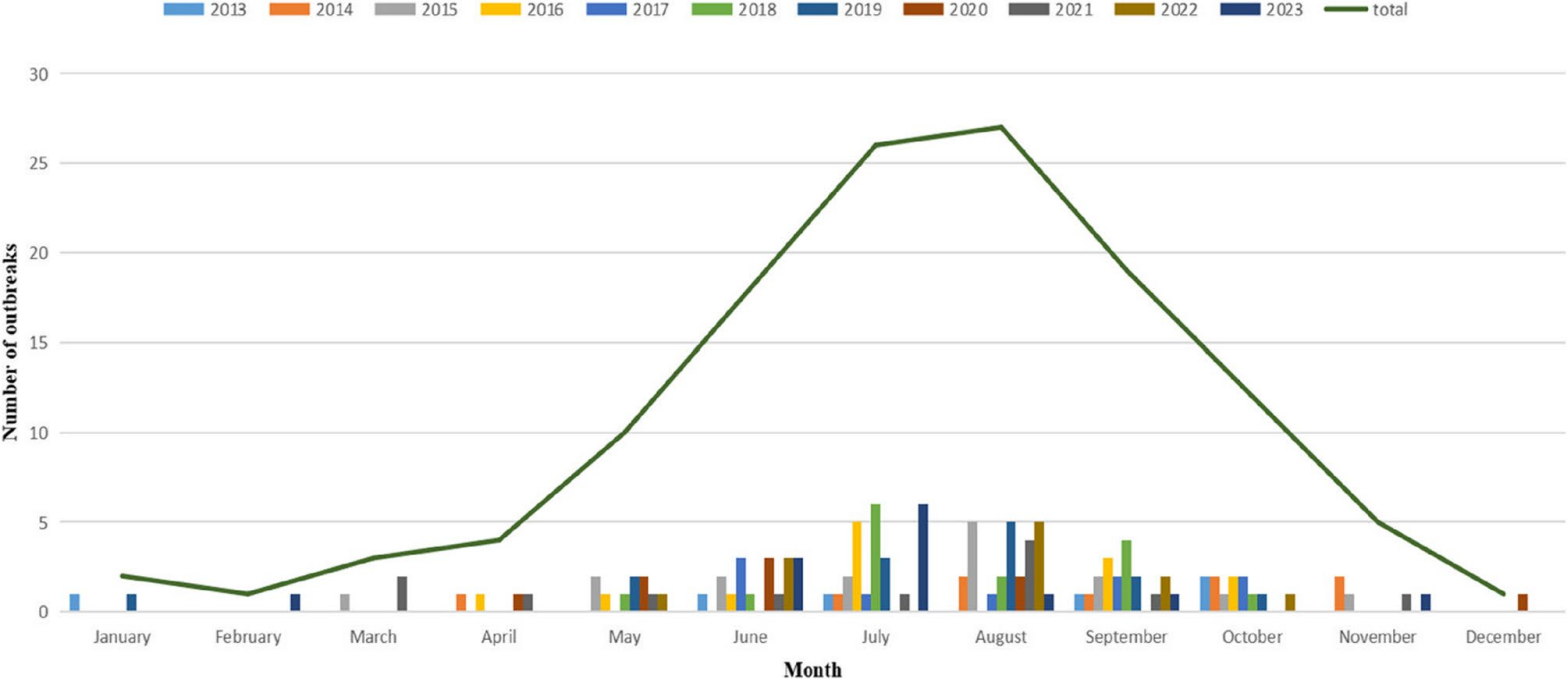
Year-month distribution of foodborne disease outbreaks in Jiaxing City, 2013–2023

During the study period, 128 FBDOs, which involved 900 cases, 117 hospitalizations, and one death, were reported through the FDOSS (Table 1). The trend in the number of reported FBDOs over time was not statistically significant (Cox-Staurt trend test, *p* = 0.69), with the highest in 2015 (16 outbreaks, 12.50%) and the lowest in 2013 (6 outbreaks, 4.69%). The trend in the number of cases involved in FBDOs over time was not statistically significant (Cox-Staurt trend test, *p* = 0.38). The average number of outbreaks and cases per year was 12 and 82, respectively, with an average of 7.03 cases reported per outbreak. Among the seven county-level cities, Tongxiang (25 outbreaks; 19.53%) reported the largest number of outbreaks, whereas Nanhu (11 outbreaks; 8.59%) reported the smallest. Outbreaks had clear seasonal characteristics, with 87.50% (112/128) occurring between May and October (Fig. 1). August (26 outbreaks, 21.09%) was the peak period for outbreaks in each year since 2013.

**Table 1:** Annual distribution of outbreaks in Jiaxing City from 2013 to 2023

Year	Outbreaks		Cases		Hospitalizations		Average number of cases per outbreak
	Number	%	Number	%	Number	%	
2013	6	4.69	120	13.33	48	41.03	20.00
2014	9	7.03	62	6.89	5	4.27	6.89
2015	16	12.50	169	18.78	9	7.69	10.56
2016	13	10.16	100	11.11	11	9.40	7.69
2017	9	7.03	48	5.33	5	4.27	5.33
2018	15	11.72	89	9.89	10	8.55	5.93
2019	14	10.94	111	12.33	16	13.68	7.93
2020	9	7.03	43	4.78	3	2.56	4.78
2021	12	9.38	66	7.33	6	5.13	5.5
2022	12	9.38	35	3.89	0	0.00	2.92
2023	13	10.16	57	6.33	4	3.42	4.38
Total	128	100.00	900	100.00	117	100.00	7.03

### Etiology

**Fig. 2:**
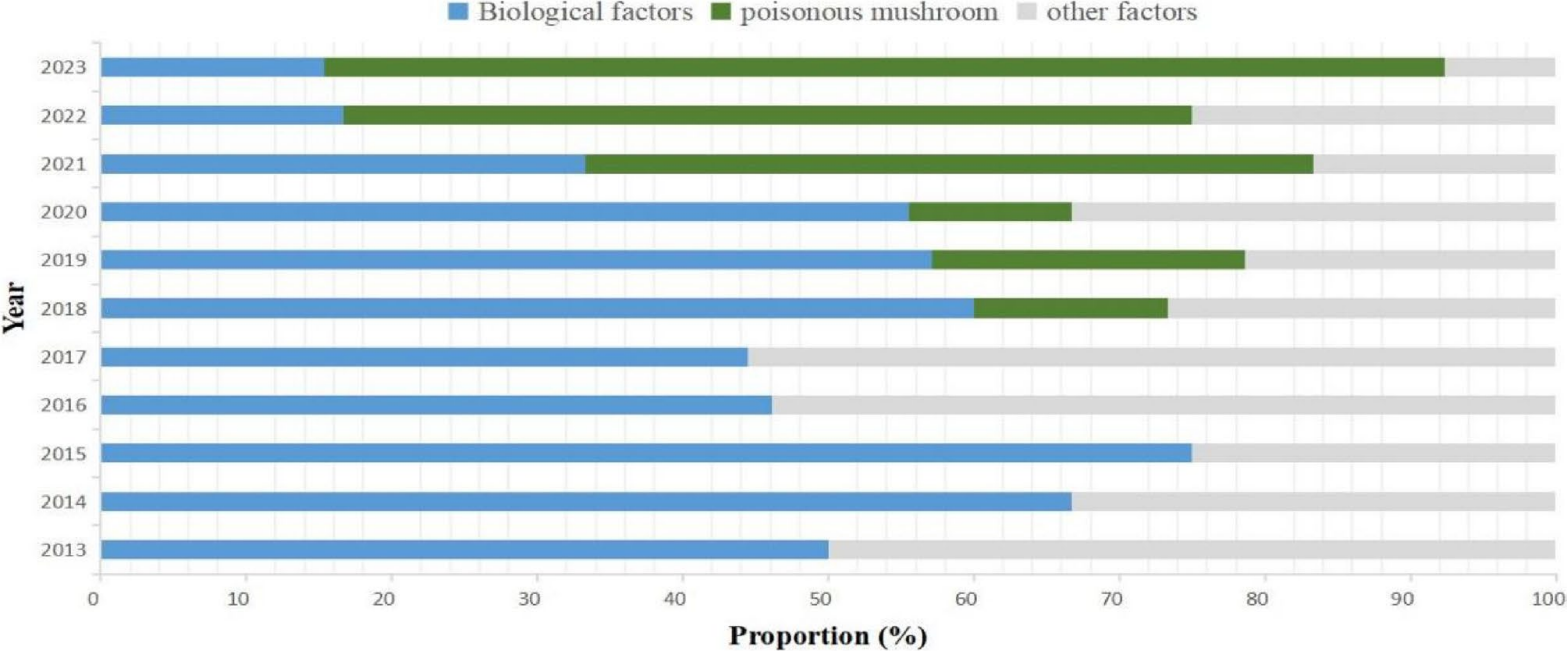
The changing trends in the composition of etiology in the FBDOs in Jiaxing City, 2013–2023

The factors responsible for 78.91% of the outbreaks reported from 2013 to 2023 were identified, and the proportion of outbreaks caused by different pathogens varied greatly (Table 2). Bacteria accounted for the highest number of outbreaks for which causative pathogens were identified (58 outbreaks, 623 cases), followed by poisonous mushrooms (28 outbreaks, 70 cases). Among the bacterial pathogens responsible for FBDOs, *V. parahaemolyticus* accounted for the highest proportion (62.07%, 36/58), followed by *Salmonella* (20.69%, 12/58). Serotyping was conducted in 36 outbreaks caused by *V. parahaemolyticus*, and the O3:K6 serotype accounted for the largest proportion (33.33%, 12/36). The most common serotype in outbreaks caused by *Salmonella* was *Salmonella* Enteritidis (50.00%, 6/12). The most common cause of outbreak-associated cases was *V. parahaemolyticus* (418 cases, 46.44%), followed by poisonous mushrooms (70 cases, 7.78%) and *Salmonella* (41 cases, 4.56%). Poisonous mushrooms accounted for a total of 28 outbreaks and 70 cases. Among the species of poisonous mushrooms, *Chlorophyllum molybdites* accounted for the highest proportion (75.00%, 21/28), followed by *Amanita rimosa* (3.57%, 1/28) and *Russula japonica Hongo* (3.57%, 1/28). And five FBDOs caused by poisonous mushrooms could not be identified with specific species. From 2018 to 2023, the proportion of outbreaks caused by poisonous mushrooms increased from 22.22 to 76.92%. Since 2021, poisonous mushrooms had become the predominant causative factors of FBDOs in Jiaxing City (Fig. 2). The proportions of other factors, such as plant toxins, noroviruses, animal toxins, and chemical agents, were relatively low, accounting for 6.25%, 2.34%, 1.56%, and 1.56% of the outbreaks, respectively.

**Table 2:** Etiological distribution of outbreaks in Jiaxing City from 2013 to 2023

Etiology	Outbreaks		Cases		Hospitalizations		Hospitalization rate(%)
	Number	%	Number	%	Number	%	
Bacterial	58	45.31	623	69.22	85	72.65	13.64
*V. parahaemolyticus*	36	28.13	418	46.44	81	69.23	19.38
*Salmonella*	12	9.38	41	4.56	3	2.56	7.32
*Diarrheagenic Escherichia coli*	2	1.56	37	4.11	0	0.00	0.00
*Bacillus cereus*	1	0.78	9	1.00	0	0.00	0.00
*Proteusbacillus vulgaris*	2	1.56	28	3.11	0	0.00	0.00
*Aeromonas*	1	0.78	22	2.44	0	0.00	0.00
*others*	4	3.13	68	7.56	1	0.85	1.47
Virus	3	2.34	20	2.22	0	0.00	0.00
*Norovirus*	3	2.34	20	2.22	0	0.00	0.00
Poisonous mushroom	28	21.88	70	7.78	18	15.38	25.71
*Chlorophyllum molybdites*	21	16.41	52	5.78	9	7.69	17.31
*Amanita rimosa*	1	0.78	2	0.22	2	1.71	100.00
*Russula japonica Hongo*	1	0.78	2	0.22	0	0.00	0.00
*Unidentified*	5	3.91	14	1.56	7	5.98	50.00
Plant toxins	8	6.25	27	3.00	1	0.85	3.70
*Undercooked Phaseolus*	1	0.78	6	0.67	0	0.00	0.00
*Bitter bottle gourd*	1	0.78	2	0.22	0	0.00	0.00
*Narcissus stem and leaves*	2	1.56	7	0.78	0	0.00	0.00
*Moldy sweet potatoes*	1	0.78	5	0.56	1	0.85	20.00
*Others*	3	2.34	7	0.78	0	0.00	0.00
Animal toxins	2	1.56	33	3.67	1	0.85	3.03
*Histamine*	2	1.56	33	3.67	1	0.85	3.03
Chemical agents	2	1.56	14	1.56	3	2.56	21.43
*Nitrite*	1	0.78	4	0.44	0	0.00	0.00
*Tung oil*	1	0.78	10	1.11	3	2.56	30.00
Unknown etiology	27	21.09	113	12.56	12	10.26	10.62
Total	128	100.00	900	100.00	117	100.00	13.00

### Setting

A total of 128 outbreak settings were reported in the data (Table 3), and the majority of outbreaks occurred in households, restaurants, and rural banquets, which accounted for 57.03%, 16.41%, and 10.16% of the total number of reported settings, respectively. Households were responsible for the highest number of cases (220, 24.44%), followed by restaurants (198, 22.00%) and staff canteens (190, 21.11%). Although households played an important role in the occurrence of outbreaks, staff canteens were responsible for the highest number of hospitalizations (47, 40.17%).

**Table 3:** Setting distribution of outbreaks in Jiaxing City from 2013 to 2023

Setting	Outbreaks		Cases		Hospitalizations	
	Number	%	Number	%	Number	%
Household	73	57.03	220	24.44	32	27.35
Restaurant	21	16.41	198	22.00	8	6.84
Rural banquet	13	10.16	174	19.33	20	17.09
Staff canteen	10	7.81	190	21.11	47	40.17
Street stall	2	1.56	21	2.33	3	2.56
Retail food outlets	3	2.34	13	1.44	1	0.85
Social meal delivery company	1	0.78	28	3.11	1	0.85
School canteen	1	0.78	26	2.89	0	0.00
Other locations	4	3.13	30	3.33	5	4.27
Total	128	100.00	900	100.00	117	100.00

### Causative foods

The causative foods were confirmed in 92 (71.88%, 92/128) outbreaks, and poisonous mushrooms (28, 21.88%) were the most common food responsible for FBDOs, followed by aquatic products (17, 13.28%), meat and meat products (13, 10.16%), and mixed foods (13, 10.16%) (Table 4). The proportion of FBDOs caused by aquatic products (159 cases, 17.67%) was the highest, followed by meat and meat products (157 cases, 17.44%) and mixed food (154 cases, 17.11%). Meat and meat products were responsible for the most hospitalizations (48 hospitalizations, 41.03%), followed by aquatic products (22 hospitalizations, 18.80%) and poisonous mushrooms (14 hospitalizations, 11.97%).

**Table 4:** Food distribution of outbreaks in Jiaxing City from 2013 to 2023

Food	Outbreaks		Cases		Hospitalizations	
	Number	%	Number	%	Number	%
Poisonous mushroom	28	21.88	70	7.78	14	11.97
Aquatic products	17	13.28	159	17.67	22	18.80
Meat and meat products	13	10.16	157	17.44	48	41.03
Mixed food	13	10.16	154	17.11	8	6.84
Toxin plants	8	6.25	27	3.00	1	0.85
Baked food	3	2.34	13	1.44	1	0.85
Vegetables and its products	3	2.34	13	1.44	5	4.27
Egg and its products	2	1.56	4	0.44	0	0.00
Bean products	2	1.56	13	1.44	0	0.00
Flour products	1	0.78	9	1.00	0	0.00
Fruit and its products	1	0.78	2	0.22	0	0.00
Beverage	1	0.78	13	1.44	0	0.00
Unknown food	36	28.13	266	29.56	18	15.38
Total	128	100.00	900	100.00	117	100.00

### Settings and etiological factors

**Fig. 3:**
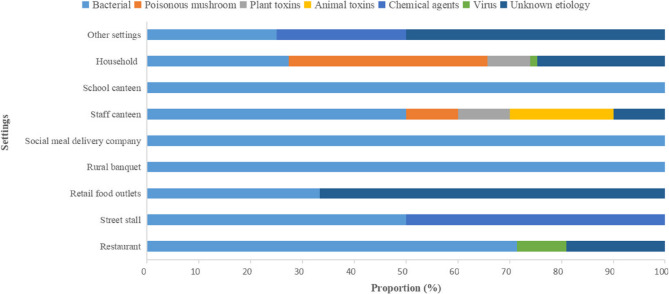
Setting-etiology distribution of FBDOs in Jiaxing City, 2013–2023

There was a statistical correlation between settings and etiological factors (χ^2^ = 125.94, *p* < 0.001). The settings of outbreaks differed in relation to the pathogenic factors (Fig. 3). In the 58 bacterial FBDOs, the most important setting was households (20 outbreaks, 34.48%), followed by restaurants (15 outbreaks, 25.86%) and rural banquets (13 outbreaks, 22.41%). Among the different pathogens responsible for bacterial FBDOs, *V. parahaemolyticus* mainly caused outbreaks in restaurants (33.33%, 12/36) and rural banquets (33.33%, 12/36), whereas the majority of *Salmonella* outbreaks occurred in households (91.67%, 11/12). Outbreaks caused by poisonous mushrooms and toxic plants occurred mainly in households, accounting for 96.43% (27/28) and 75.00% (6/8) of the total, respectively. Viruses caused a total of three outbreaks, two in restaurants and one in households.

### Foods and etiological factors

There was a statistical correlation between foods and etiological factors (χ^2^ = 64.22, *p* < 0.001). The results showed that the causative foods tended to differ in relation to the pathogenic factors. The main cause of outbreaks caused by poisonous mushrooms was *Chlorophyllum molybdites* (75.00%, 21/28). *V. parahaemolyticus* was the main pathogenic factor in outbreaks attributable to aquatic products and meat and meat products, accounting for 64.71% (11/17) and 30.77% (4/13) , respectively. The meat products that caused *V. parahaemolyticus* infection were mainly cooked meats, including cold dish duck offal, chicken salad, and cold dish pig ears, which were not reheated before consumption. The foods that caused *Salmonella* outbreaks were mainly meat and meat products (25.00%, 3/12) and aquatic products (16.67%, 2/12). The meat and meat products that caused outbreaks were mainly ready-to-eat cold-cooked meats, and the causative aquatic products were mainly not-fully-cooked aquatic products. Animal toxins were only found in aquatic products (100%, 2/2), and the pathogenic factor was histamine poisoning in green catfish. Plant toxins were mainly found in the stems and leaves of *Narcissus* (25.00%, 2/8), moldy sweet potato (12.50%, 1/8), *Phaseolus* (12.50%, 1/8), and bottle gourds (12.50%, 1/8). Chemical agents such as tung oil (50.00%, 1/2) and nitrites (50.00%, 1/2) were the main chemical pathogenic factors that caused outbreaks. Outbreaks caused by tung oil and nitrites were mainly caused by misuse of condiments.

## Discussion

From 2013 to 2023, 128 FBDOs involving 900 cases and 117 hospitalizations were reported in Jiaxing City. The gradual refinement of the monitoring and reporting systems has greatly enhanced the ability to identify FBDOs and provide early warnings regarding these outbreaks. With the strengthening of food safety supervision and implementation of various forms of food safety publicity activities, the scale of outbreaks has been shrinking. However, in comparison with 2013, the number of reported FBDOs in 2023 had increased, indicating that food safety issues still require greater attention from the government and social organizations.

Although they occurred throughout the year, FBDOs in Jiaxing City showed seasonality; summer and autumn were the peak periods of FBDOs, and monitoring results from other regions in China also revealed this characteristic [13, 14]. Summer and autumn are the high-risk periods for foodborne bacterial diseases. Previous studies have shown that warm weather accelerates the reproduction of bacteria and increases the risk of bacterial FBDOs [8, 15]. *V. parahaemolyticus* and *Salmonella* were the main biological factors associated with the outbreaks in Jiaxing City. *V. parahaemolyticus* infection was common in the eastern coastal areas of China [16]. In Jiaxing City, the temperature and humidity are high from June to August, allowing rapid multiplication of microorganisms. Therefore, the prevention and control of bacterial outbreaks in the summer and autumn, especially in August, requires attention.

Analysis of the correlations between pathogens and foods showed that *V. parahaemolyticus* outbreaks were often linked to aquatic products and meat and meat products. Similarly, *Salmonella* outbreaks were often linked to meat and meat products. Studies from the European Union and the United States showed that seeded vegetables, eggs, poultry, beef, and pork were responsible for *Salmonella* outbreaks [6, 17]. In Jiaxing, the consumption of aquatic and meat products is relatively high. Thus, food provided at dining events in hotels, restaurants, and rural banquets often contains aquatic products as well as meat and meat products. The ingestion of undercooked aquatic products and cross-contamination during food processing are the main reasons for outbreaks of foodborne diseases caused by *V. parahaemolyticus* and *Salmonella.* Cross-contamination in food processing may involve the mixing of raw and cooked foods as well as the use of shared containers [18]. Targeted health education, supervision, and management measures should be implemented for key populations, locations, and high-risk periods to reduce the probability of FBDOs caused by *V. parahaemolyticus* and *Salmonella*.

The monitoring data evaluated in this study suggested that the incidence of mushroom poisoning has increased in recent years. Between 2018 and 2023, the proportion of outbreaks triggered by poisonous mushrooms rose from 22.22% to 76.92%. A previous study conducted in other regions showed that poisonous mushrooms were responsible for the most deaths [19]. The detailed species of poisonous mushrooms that caused FBDOs were *Chlorophyllum molybdites*,* Amanita rimosa* and *Russula japonica Hongo*. *Chlorophyllum molybdites* accounted for the majority of poisonous mushrooms that caused FBDOs in Jiaxing. The study conducted in southern China showed that species of the Amanita genus were responsible for 64.70% of poisoning outbreaks, 78.05% of poisoning cases, and 70.49% of deaths [20]. There are many types of poisonous mushrooms, and factors such as terrain and climate contribute to significant differences in their growth across various regions. It is difficult to identify the causative species because of absence of relevant mushrooms and ingestion of multiple mushrooms. Accurate and prompt species identification is crucial in the diagnosis and treatment process. More effort and cooperation is needed from administrative agencies, epidemiologists, doctors, and mycologists to increase the identification rate [21].

FBDOs caused by poisonous mushrooms in Jiaxing often occurred in households, consistent with the results of other studies [22–25]. Outbreaks caused by poisonous mushrooms can be attributed to two main reasons. First, because of the low awareness of food safety, local residents collect wild mushrooms from grasslands, roadsides, or other sites and cook and eat them in households. Poisonous mushrooms and their toxins were also the main pathogenic factors causing the high numbers of FBDOs in Yunnan Province, China [26]. The second reason is related to the association between the upward trend in outbreaks caused by poisonous mushrooms and the influx of migrants and their eating habits, since these migrants often collected and consumed poisonous mushrooms because of their similarity to other non-toxic mushrooms. Thus, health education should focus on key knowledge points, such as not picking, purchasing, processing, or eating unfamiliar wild mushrooms. To this end, the existing forms of advertisement, including radio or television, should be fully utilized to disseminate this knowledge in high-risk areas and among migrants and rural residents to enhance food safety awareness and prevent wild mushroom poisoning.

Households accounted for the highest proportion of FBDOs in Jiaxing City, consistent with research results from other regions of China [27, 28]. The proportion of FBDOs in households showed an upward trend during the study period. This may be related to potential food safety hazards such as handling of leftovers, cross-contamination of raw and cooked food, and kitchen hygiene [18]. During implementation of food safety publicity for community residents, the correct methods of food selection, processing, and preservation should focus on reducing the food safety risks caused by homemade food [29]. Although rural banquets had a relatively low proportion of outbreaks, they involved a larger number of cases and hospitalizations. Once FBDOs occur, they are prone to have substantial negative social effects. Therefore, for rural banquets, the establishment of fixed banquet venues, supervision and training of practitioners, and other measures should be implemented to reduce the risk of outbreaks.

This study had some limitations. First of all, during the COVID-19 pandemic, the frequency of dining out decreased, while the frequency of household dining increased significantly. Hence, the proportion of household would be higher than the normal years, leading to changes in the composition of factors caused FBDOs. Incomplete information may have been recorded in the monitoring system during epidemiological investigations of outbreaks because of a lack of cooperation from the cases or the dining venue manager. Moreover, the patients’ inability to recall the consumed food and other factors, such as administration of antibiotics before the collection of biological samples, may have limited the ability to identify the causes of outbreaks and their pathogenic factors. In addition, due to surveillance biases and limitations in passive reporting from hospitals, the actual number of outbreaks may be underestimated. We have taken a variety of measures to reduce possibility of underreporting. Firstly, we have provided training and guidance to medical institution and CDCs of all levels every year to improve the reporting of FBDOs. Secondly, we have optimized the reporting procedure and improved the accuracy of data collection. It is worth mentioning that the function of cluster warnings has been added in FDSRS, and the ability of identifying clusters of cases has been improved significantly. In the future, we will utilize the powerful data analysis and integration capabilities of artificial intelligence models to identify potential outbreak clues and combine multi-source data cross-validation to further improve the accuracy of outbreaks monitoring.

## Conclusions

Poisonous mushrooms and microbial pathogens are the main factors causing FBDOs. Since 2018, the proportion of FBDOs caused by poisonous mushrooms has been increasing annually. Since the general public does not have a reliable way to identify poisonous mushrooms, we recommend not picking, buying or eating wild mushrooms. Households are the most important settings for FBDOs. We recommend that advertisement of food safety knowledge should be carried out among community residents to raise their awareness. Most foodborne diseases are preventable; therefore, timely investigation, effective management, and accurate reporting of FBDOs can significantly contribute to their reduction.

## Data Availability

The data supporting the findings of this study are available from the FDOSS managed by CFSA, and these data are not publicly available.

## Abbreviations

FDOSS: Foodborne Disease Outbreak Surveillance System
FBDOs: Foodborne Disease Outbreaks
WHO: World Health Organization
FDSRS: Foodborne Disease Surveillance and Reporting System
CFSA: China National Center for Food Safety Risk Assessment
CDC: Center for Disease Control and Prevention
